# Prediction of gait trajectories based on the Long Short Term Memory neural networks

**DOI:** 10.1371/journal.pone.0255597

**Published:** 2021-08-05

**Authors:** Abdelrahman Zaroug, Alessandro Garofolini, Daniel T. H. Lai, Kurt Mudie, Rezaul Begg

**Affiliations:** 1 Institute for Health and Sport, Victoria University, Melbourne, Victoria, Australia; 2 College of Engineering and Science, Victoria University, Melbourne, Victoria, Australia; 3 Defence Science and Technology Group, Melbourne, Victoria, Australia; Fuzhou University, CHINA

## Abstract

The forecasting of lower limb trajectories can improve the operation of assistive devices and minimise the risk of tripping and balance loss. The aim of this work was to examine four Long Short Term Memory (LSTM) neural network architectures (Vanilla, Stacked, Bidirectional and Autoencoders) in predicting the future trajectories of lower limb kinematics, i.e. Angular Velocity (AV) and Linear Acceleration (LA). Kinematics data of foot, shank and thigh (LA and AV) were collected from 13 male and 3 female participants (28 ± 4 years old, 1.72 ± 0.07 m in height, 66 ± 10 kg in mass) who walked for 10 minutes at preferred walking speed (4.34 ± 0.43 km.h^-1^) and at an imposed speed (5km.h^-1^, 15.4% ± 7.6% faster) on a 0% gradient treadmill. The sliding window technique was adopted for training and testing the LSTM models with total kinematics time-series data of 10,500 strides. Results based on leave-one-out cross validation, suggested that the LSTM autoencoders is the top predictor of the lower limb kinematics trajectories (i.e. up to 0.1s). The normalised mean squared error was evaluated on trajectory predictions at each time-step and it obtained 2.82–5.31% for the LSTM autoencoders. The ability to predict future lower limb motions may have a wide range of applications including the design and control of bionics allowing improved human-machine interface and mitigating the risk of falls and balance loss.

## Introduction

Prediction of gait kinematics is a useful approach to improve the operation of assistive devices (i.e. bionics) and minimise the risk of falling [1–5]. Prediction of the human gait however has been a challenging process due to the locomotor system’s high degrees of freedom that continuously change and the asymmetrical foot contact with the ground [6]. One of the most common straightforward approach for gait prediction is to combine forward dynamics with optimisation methods in which human muscle forces and limb movements are determined by minimising a cost function [7,8]. The method, however, requires a long computational time, and it is highly dependent on the measured data [9,10]. In order to achieve efficient computational time and to set the optimisation parameters without relying on the measured data, inverse dynamics along with optimisation methods are implemented to predict the human walking trajectories [11]. Ren *et al*. [12] predicted all segments motion and ground reaction forces from the average gait forward velocity, double stance duration and the period of the gait cycle. Although those methods are able to capture the gait features, they idealise the human motions and were unable to generalise the gait trajectories [13,14].

The emergence of inexpensive wearable sensors such as Inertial Measurement Units (IMUs) [15,16] have posed new challenges (i.e. increase in dimensionality) and opportunities (i.e. new insights) to the analysis of the human movement [17] outside laboratory settings [4]. In response to these challenges, a new set of Machine Learning (ML) algorithmic models have been widely adopted by biomechanists [17,18]. The ML algorithms are a subfield of Artificial Intelligence (AI) concerned with the establishment of computer programmes that learn patterns from data [19]. Computational techniques related to ML have been successful in solving several aspects of biomechanics gait research problems [20,21], such as the gait classification [22–24], joint angle prediction [25] and energy expenditure minimisation in lower limb exoskeletons [26]. Tanghe *et al*. have applied the Probabilistic Principal Component Analysis (PPCA) to predict the future lower limb joint kinematics and achieved an error rate between 4.5–12.5% [27]. The data however were collected at an imposed speeds (2 and 5 km.h^-1^) and the error was calculated at 3 points only in the gait cycle (10, 50 and 100%) [27].

One of the most utilised algorithms for the human movement prediction are the Artificial Neural Networks (ANNs) [17,28,29]. A class of ANN known as deep learning (inspired by the structure and function of the brain) [30], were found to be insightful in human activity classification [31–33], gait pattern recognition [34] and the improvement of user intention detection in wearable assistive devices (i.e. bionics) [35–38]. It was also applied in regression tasks such as the prediction of lower limb joint angles from the Angular Velocity (AV) and Linear Acceleration (LA) of foot and shank segments [39]. Gholami *et al*. implemented Convolutional Neural Networks (CNN) to predict the lower limb joint angles from the foot LA data and achieved between 6.5–11.1% error rate [40]. Nonetheless, the developed ANNs in the literature were predicting the gait trajectories (i.e. knee angles) from an independent variable (i.e. foot LA) and it was not implemented to predict future gait trajectories of the same independet variable.

The kinematics of the lower limbs are the means by which powered exoskeletons are controlled, falls are prevented and abnormal gaits are identified [1–4] The authors are not aware of previous work that investigated sequential ML models such as LSTM neural networks to predict future gait trajectories at preferred walking speed (PWS) and imposed walking speed. The LSTM neural networks are an ANN architecture known for modelling time-series information [41,42]. The LSTM neural networks have proven wide success in modelling human movement data such as the lower limb kinematics prediction [43] neurodegenerative disease diagnosis [44], gait event detection [45,46] and falls recognition [47]. The aim of this work was to develop and compare four standard LSTM architectures (Vanilla, Stacked, Bidirectional and Auto-encoders) for the prediction of future lower limb trajectories, i.e. foot AV, shank AV, thigh AV, foot LA, shank LA and thigh LA. In our previous paper [48], we found that LSTM autoencoder (i.e. LSTM architecture) is able to predict the future gait trajectories at an imposed speed. This work further investigates different LSTM architectures in predicting future gait kinematics when individuals walk at PWS and an imposed speed.

## Materials and methods

### Study participants

Walking data were collected from 13 male and 3 female participants (28 ± 4 years old, height 1.72 ± 0.07 m, mass 66 ± 10 kg) who walked for 10 minutes at their PWS (4.34 ± 0.43 km∙h^-1^) and at an imposed speed (5 km∙h^-1^, 15.4% ± 7.6% faster) on a 0% gradient treadmill. Trials were randomised and one participant’s data (male) was omitted due to incomplete data. The PWS was calculated by starting the treadmill at 3 km∙h^-1^, then it was gradually increased until the participant says “this is comfortable”. It was again increased until an uncomfortable speed (i.e fast walking) was reached. After that, speed was gradually decreased until the participant says “this is comfortable”. An average value was then calculated from the two comfortable recorded speeds to represent the PWS [49]. Each trial was started with a 2 minutes familiarisation session for treadmill walking. Ethics approval was granted by the Victoria University Human Research Ethics Committee (ID HRE18-230) and the Department of Defence and Veterans’ Affairs Human Research Ethics Committee (Protocol 852–17). All participants signed a consent form and volunteered freely to participate.

### Gait analysis

A set of 30 retroreflective markers were attached to each participant in the form of clusters [39]. Each cluster comprised of a group of individual markers that represent a single body segment (e.g. shank). This include left and right foot clusters (3 markers), left shank cluster (5 markers), right shank cluster (5 markers), left thigh cluster (5 markers), right thigh cluster (5 markers) and pelvis cluster (4 markers). The 3D position of each cluster was tracked using a 14 camera motion analysis system (Vicon Bonita, Version 2.8.2) recording at 250Hz. Before capturing the dynamic trials, a static pose (1 second) was recorded where an additional 8 retroreflective markers were placed on anatomical landmarks (e.g. lateral femur medial epicondyle) identified by palpation [50–52]. The static pose was used to calibrate the position and orientation of the lower body skeletal system [53].

### Kinematic walking profiles

**Fig 1:**
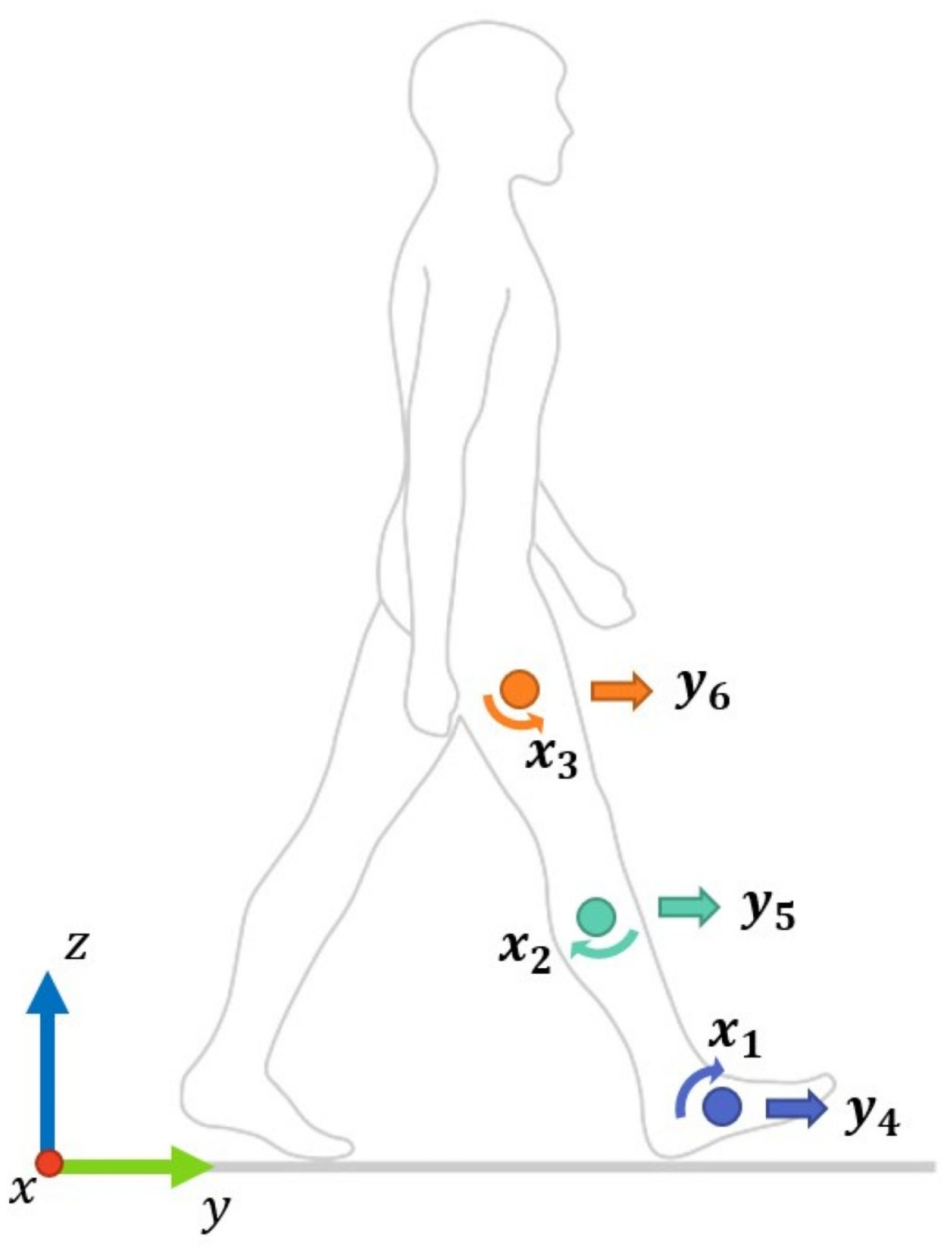
Definition of sagittal plane movements as well as the (X,Y) coordinates. Sagittal plane movements included the rotation around the X-axis (i.e., AV) and the translation along the Y-axis (i.e., LA).

**Fig 2:**
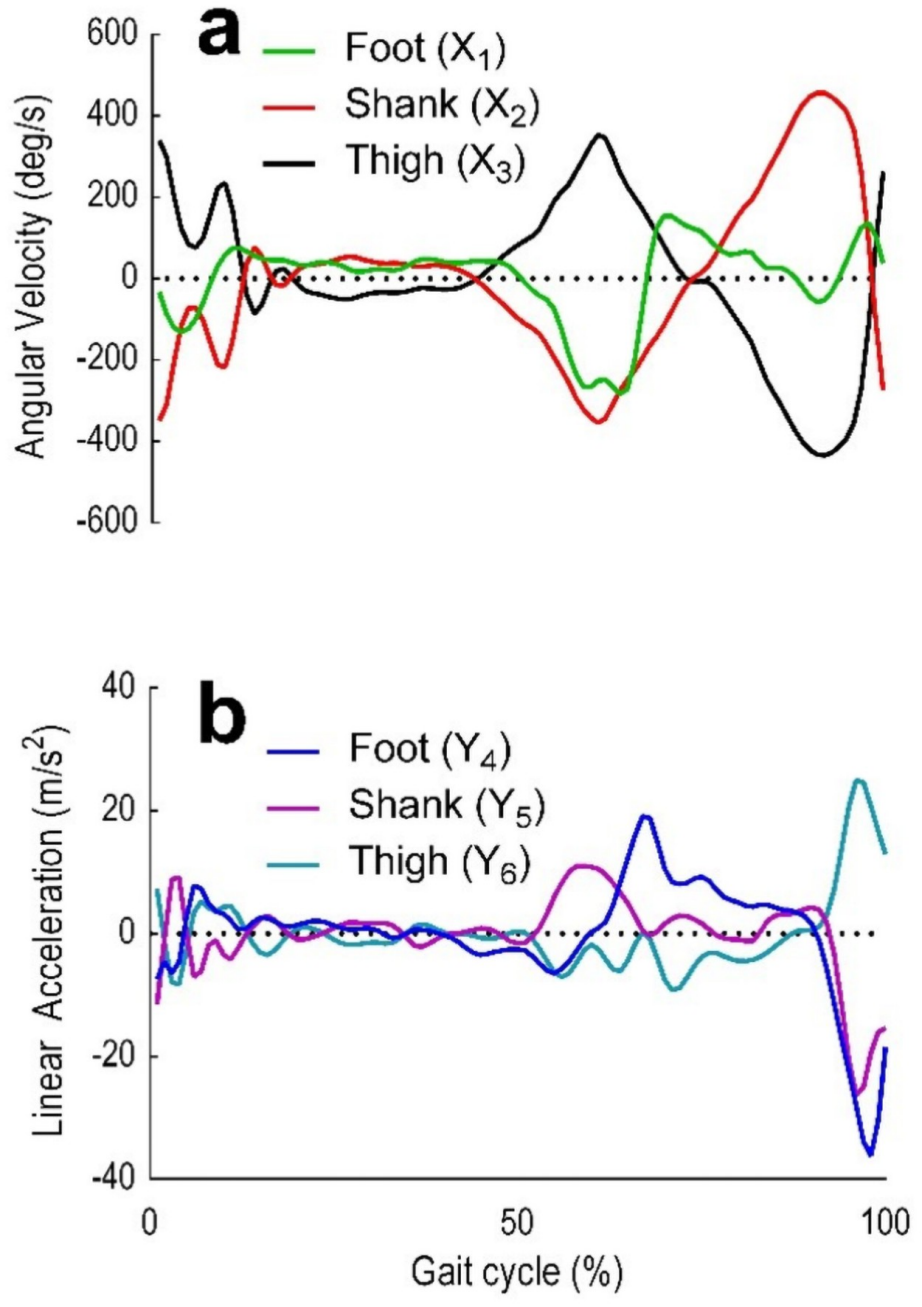
Foot, shank and thigh sagittal plane AV and LA. Those were selected as the model’s independent variables predominantly because primary motions of the human movement are flexion and extension in the sagittal plane (6). (a) Foot (X_1_), shank (X_2_) and thigh (X_3_) AV. (b) Foot (Y_1_), shank (Y_2_) and thigh (Y_3_) LA.

Recorded 3D positional data were processed using Visual 3D (C-motion, Inc, Version 6) to compute LA and AV for the thigh, shank, and foot segments of the right limb [48]. LA and AV were then interpolated with a least-squares fit on a 3^rd^ order polynomial and filtered using a lowpass digital filter with a 15Hz cut-off frequency [54,55]. A stride was defined as the interval between two successive heel strikes of the right foot [56]. Outlier strides (i.e. bad strides) were labelled as bad and excluded from the final time series data. The final time series data were downsampled to 50 Hz (to accelerate LSTM computational time) [43] and normalised with z-scores using Matlab (Mathworks, Inc, R2020a). The sagittal plane kinematics included the translation along the Y-axis (i.e. LA) and the rotation along the X-axis (i.e. AV), and were used for LSTM prediction, resulting in six predictor variables, (i) *X*_1_ foot AV (ii) *X*_2_ shank AV (iii) *X*_3_ thigh AV (iv) *Y*_4_ foot LA (v) *Y*_5_ foot LA and (vi) *Y*_6_ foot LA (Figs 1 and 2).

### Datasets

Processed time series data (10,500 strides) were combined to include the two walking speeds. The total dataset comprised of 10,500 strides from 15 participants that included 6 kinematic feature variables (*X*_1_, *X*_2_, *X*_3_, *Y*_4_, *Y*_5_, *Y*_6_). The dataset was divided into training set from 14 participants and a testing set from 1 participant. To evaluate the generalisation capability using leave-one-out cross validation, the testing set was created for each participant comprised of 75 timesteps (i.e. 1.5s of the gait cycle) and the 6 kinematic feature variables (Table 1). A single timestep is equivalent to 0.02s (i.e. 1/50Hz). The 75 timesteps was selected based on the average stride time and was selected from the first right foot strike following the familiarisation session.

**Table 1:** Train-test split datasets.

Validation protocol	Number of participants	
	Training	Testing
**leave-one-out**	14	1, 75 timesteps only

The model was trained and tested using leave-one-out cross validation. At each epoch, the training set doesn’t contain all trials of the tested participant.

### Time series transformation to a supervised learning problem

**Fig 3:**
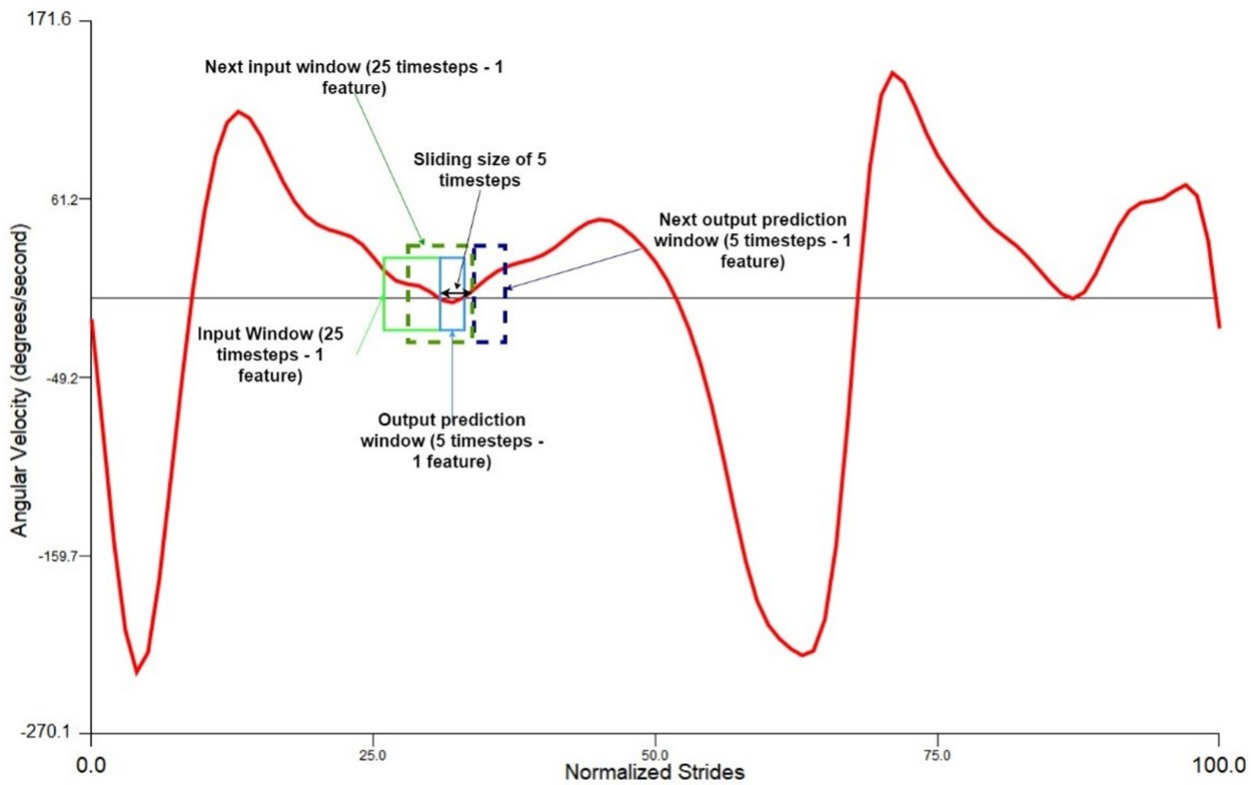
Sliding window demonstration on 1 feature. The graph shows the sliding window operation on the foot angular velocity (*X*_1_). In this paper, the input/output window comprises of 6 features.

The T*F (i.e. Timesteps*Features) data structure was transformed into S*T*F (i.e. Samples*Timesteps*Features) structure (Fig 3) [48]. One sample is a one window that consists of multiple timesteps and the 6 features. A single timestep is equivalent to 0.02s (i.e. 1/50Hz). The training input data was transformed from 614,083 timesteps and 6 features into 122,811 samples and inside of each sample are 25 timesteps and 6 features. While the corresponding output training data was 112,811 samples and inside of each sample are 5 timesteps and 6 features. The testing input data was converted from 75 timesteps and 6 features to 15 samples, 25 timesteps and 6 features. While the corresponding output testing data was converted to 15 samples, 5 timesteps and 6 features.

### The LSTM model architectures

Regression problems are amongst the challenging tasks to ANN [57]. Due to the parameters’ initialisation (i.e. neuron weights), the bias-variance trade-off and the function approximation that may trap at a local minima [57]. As such, it is necessary to experiment with different network architectures to find the optimum solution. After determining the possibility of future trajectories in our last paper [48], this work was to determine which LSTM architecture performs the best. There was no report that was found looking into the optimum LSTM neural networks model for gait forecasting prediction. There were 4 LSTM neural network variants that have been tested in this paper. This include; (1) Vanilla LSTM neural networks, (2) Stacked LSTM neural networks, (3) Bidirectional LSTM neural networks and (4) LSTM autoencoders neural networks. A detailed description for the LSTM model as well as each architecture is provided in the S1 Appendix.

The sliding windows and the LSTM models were developed using Python 3 (Libraries: Keras, Numpy, Pandas and Scikit learn) and executed in Amazon Web Services (AWS) Elastic Computing (EC2) [58,59]. The networks were optimised using Stochastic Gradient Descent (SGD) optimisation algorithm [60–62]. Proposed by Rumelhart *et al*,. 1986 [63], the SGD algorithm aims to obtain the minimum error (MAE in this work) at each batch using the network weights and biases. Using a sparse grid-search, for all models the SGD’s learning rate was tuned to 0.07, the gradient norm was clipped to 1.0 and the momentum (for accelerating the gradient descent) was set to 0.9 [64].

### Evaluation and performance metrics

The input/output sliding window size was kept fixed throughout models [65,66]. The input window was 25 time-steps (0.5s) and the output window (future prediction—0.1s) was 5 time-steps (0.1s) for the 6 feature variables; foot (X_1_), shank (X_2_), thigh (X_3_) AV and foot (Y_4_), shank (Y_5_) and thigh (Y_6_) LA (48).

The models’ configuration (Table 2) was determined based on a sparse grid search for obtaining the least MAE on an unseen participant. All participants were tested using the leave-one-out cross validation technique. When a participant is tested all of their associated trials were removed from the training set (5k/h and PWS). In order to fascilitate model comparison, the models were compared based on the same tested participant. Due to the uniqueness of individual walking patters and the different PWS across participants [67,68], the testing set for all participants was kept fixed at 75 time-steps (starting from the foot strike).

**Table 2:** Models’ configuration for inter-subject leave-one-out cross validation test.

Models	Hidden layers	Units per layer	Epochs
**Vanilla LSTM**	1	1024	100
**Stacked LSTM**	5	256	260
**BI-LSTM**	2	1024	200
**ED-LSTM**	2	1024	200

To evaluate each model’s performance, 5 parameters were considered to calculate how closely the predicted variable trajectories $\hat{y_{j}}$ (*X*_1_, *X*_2_, *X*_3_, *Y*_4_, *Y*_5_, *Y*_6_) were to the actual variable trajectories *y*_*j*_ (*X*_1_, *X*_2_, *X*_3_, *Y*_4_, *Y*_5_, *Y*_6_) across the *n* timesteps:

1. Mean Absolute Error (MAE) given as:
$$MAE=\frac{1}{n}\;\sum_{j=1}^{n}\left|y_{j}-\;\hat{y_{j}}\right|$$ (13)
2. Mean Squared Error (MSE) given as:
$$MSE=\frac{1}{n}\;\sum_{j=1}^{n}{\left(y_{j}-\;\hat{y_{j}}\right)}^{2}$$ (14)
3. Correlation coefficient (CC) given as:
$$P=\frac{cov(y,\hat{y})}{std(y)*std(\hat{y})}$$ (15)
  Where, *std*() is the standard deviation and $cov(y,\hat{y})$ is the covariance between variables *y* and $\hat{y}$.
4. Root Mean Square Error (RMSE) given as:
$$RMSE=\sqrt{\frac{1}{n}\;\sum_{j=1}^{n}{\left(y_{j}-\;\hat{y_{j}}\right)}^{2}}$$ (16)
5. Normalised RMSE (NRMSE) (27, 40) given as:
$$NRMSE(\%)=\frac{RMSE}{max(\text{y})-\text{min}(\text{y})}$$ (17)
  Where max (y) and max (n) are the maximum and minimum values of the trial’s ground truth.

## Results

**Fig 4:**
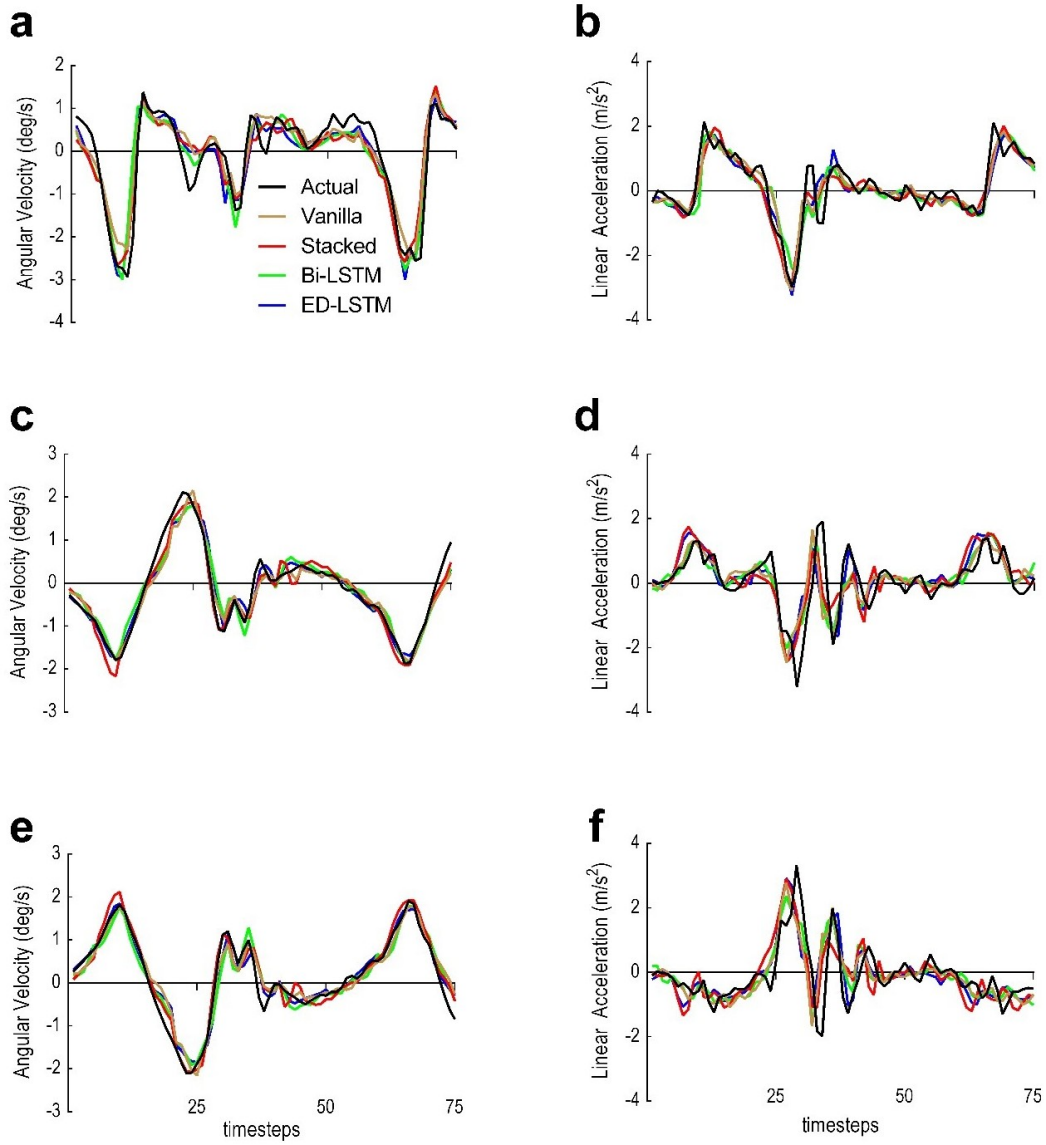
LSTM models prediction performance based on the inter-subject test for each feature vector at PWS only. Models were tested with 75 time-steps and the same participant was tested across LSTM models. Black is the actual trajectory. Brown is the Vanilla LSTM predicted trajectory. Red is the Stacked LSTM predicted trajectory. Green is the Bi-LSTM predicted trajectory. Blue is the ED-LSTM predicted trajectory. (a) Foot AV (*X*_1_). (b) Foot LA (*Y*_4_). (c) Shank AV (*X*_2_). (d) Shank LA (*Y*_5_). (e) Thigh AV (*X*_3_). (f) Thigh LA (*Y*_6_).

All models were firstly evaluated at PWS and on the same participant across 75 timesteps. Trials related to the tested participant were removed from the training set. Models performance (combined output windows) for each of the independent variables that belong to the tested participant are shown in Fig 4 for all LSTM models. A single output window (prediction horizon) is a 5 time-steps window (0.1s). All models have shown good tracking of the actual trajectories for the shank and thigh AV (Fig 4c and 4e). Poorer predictions were attained for all segment predictions based on LA.

**Fig 5:**
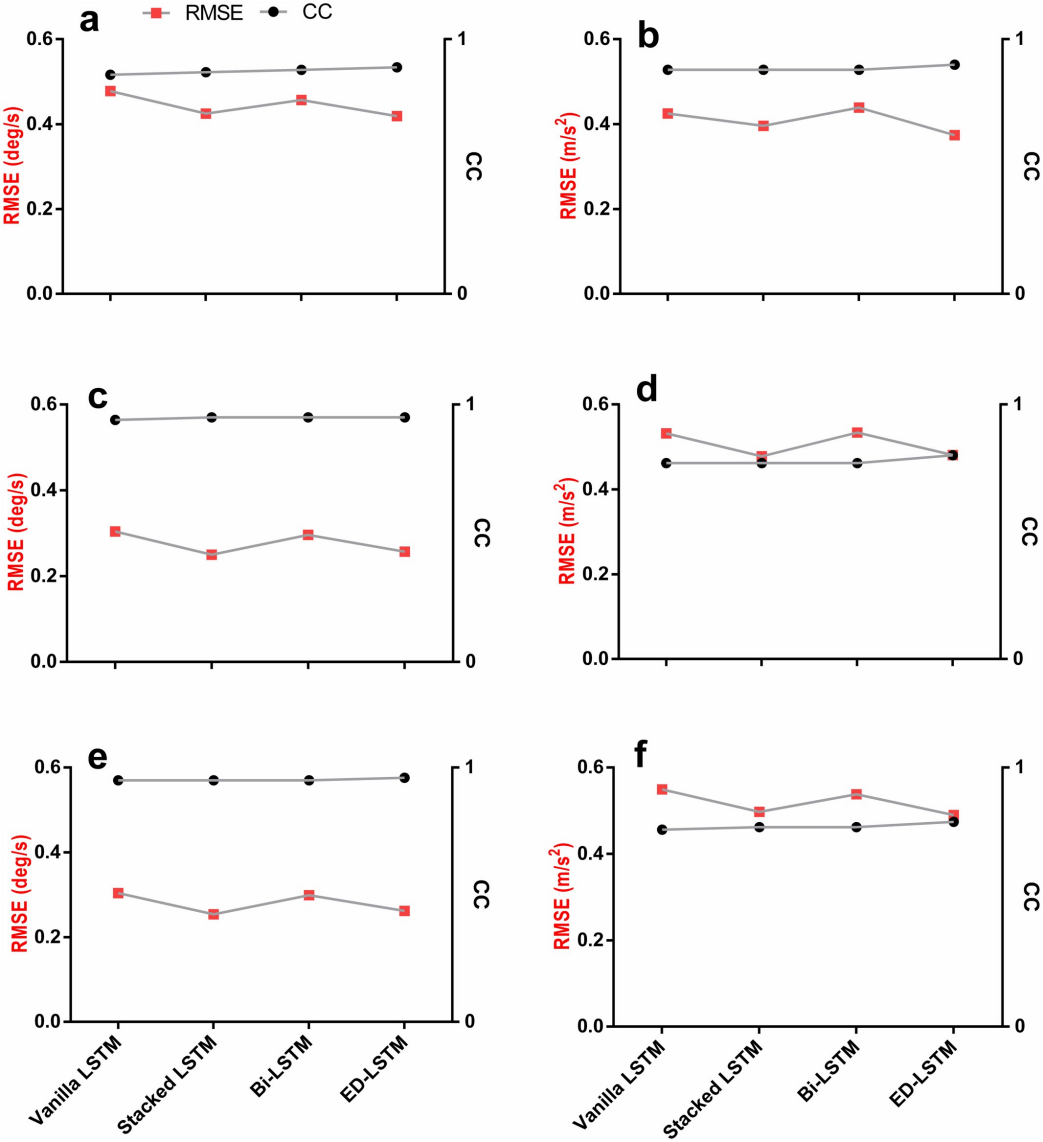
Performance comparison between LSTM models based on leave-one-out cross validation at PWS and 5km.h^-1^ for each feature vector. Red is the RMSE (Left Y-axis). Black is the CC (Right Y-axis). Wider gaps between the two error lines (CC and RMSE) means better prediction quality for the related feature vector. The Stacked and ED LSTM maintained the gap for all feature vectors. (a) Foot AV (*X*_1_). (b) Foot LA (*Y*_4_). (c) Shank AV (*X*_2_). (d) Shank LA (*Y*_5_). (e) Thigh AV (*X*_3_). (f) Thigh LA (*Y*_6_).

**Fig 6:**
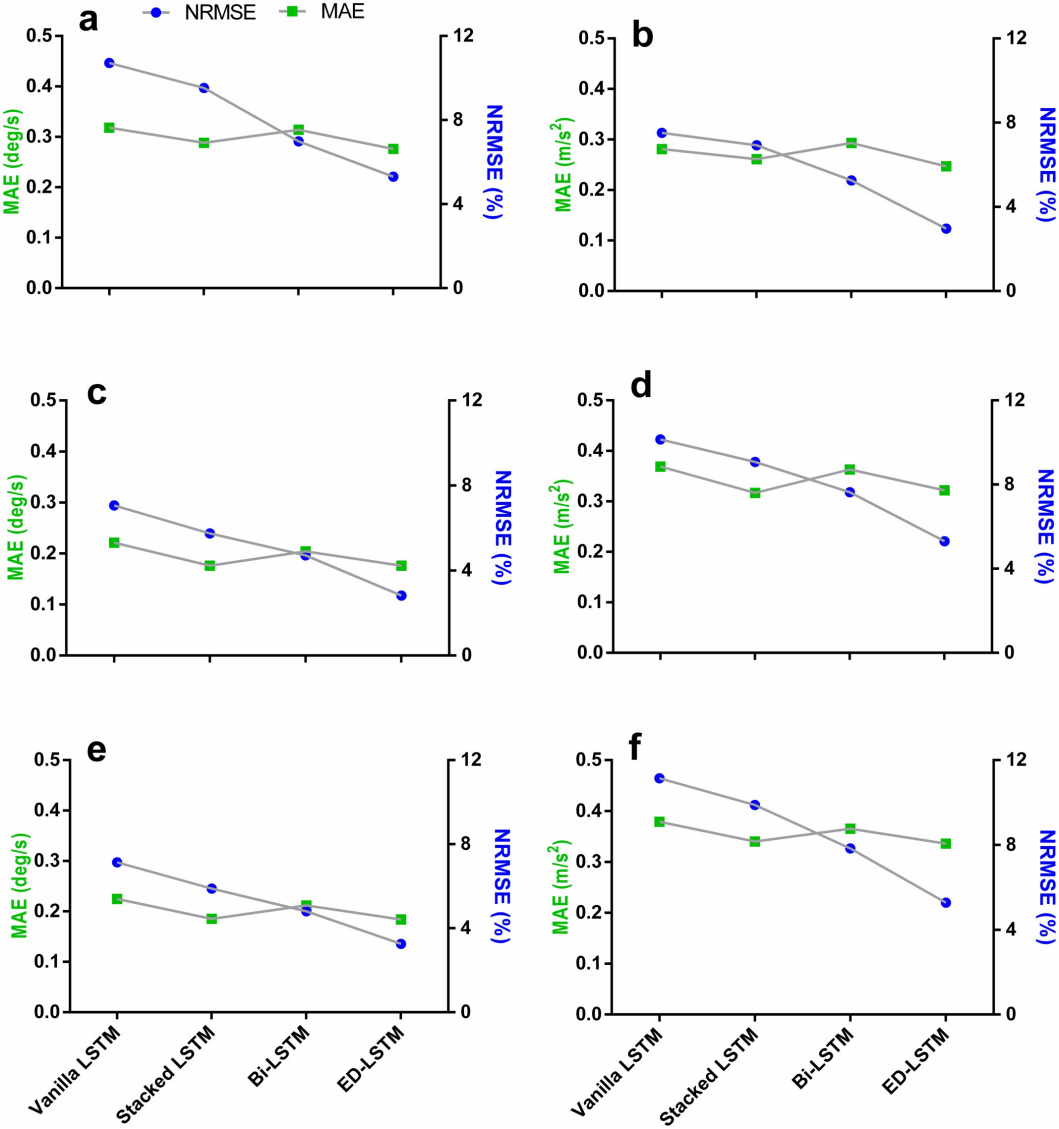
Performance comparison between LSTM models based on leave-one-out cross validation at PWS and 5km.h^-1^ for each feature vector. Green is the RMSE (Left Y-axis). Blue is the NRMSE (Right Y-axis). Lower error points for the MAE and NRMSE means a better predictive model for the related feature vector. (a) Foot AV (*X*_1_). (b) Foot LA (*Y*_4_). (c) Shank AV (*X*_2_). (d) Shank LA (*Y*_5_). (e) Thigh AV (*X*_3_). (f) Thigh LA (*Y*_6_).

Models were then evaluated at PWS and 5km.h^-1^ combined based on leave-one-out cross validation technique for each participant (see Tables 3 and 4). All models achieved good predicted trajectories for AV related to the shank (***X***_2_) and thigh (***X***_3_) (low RMSE in Fig 5c and 5e) and good vector patters for all feature vectors (High CC in Fig 5). Predicted trajectories based on the AV (MAE 0.176–0.318 deg/s) were generally less erroneous than the predicted trajectories based on the LA (MAE 0.184–0.379 m/s^2^) across all the LSTM models (see Table 3). The ED LSTM is found the best predictive model for future predictions of the lower limb kinematic trajectories at PWS and 5km.h^-1^. The wider the gap between the two points (CC and RMSE) in Fig 5 indicates a good performance achieved by the LSTM model. The Stacked and ED LSTM were the only models that maintained a good predicted LA and AV patterns (Higher CC in Fig 5—Table 5) and a more accurate predicted LA and AV trajectories (lower RMSE in Table 3 and Fig 5). The Vanilla LSTM obtained the poorest prediction results (7.06–11.14%) compared to the Stacked LSTM (5.74–9.88%), the Bi-LSTM (4.71–7.83%) and the ED-LSTM (2.82–7.91%) (Table 4 and Fig 6).

**Table 3:** Leave-one-out cross validation test error based on the MAE, MSE and the RMSE at the PWS and 5km.h^-1^ combined.

Error metric	Architecture	*X*_1_ (deg/s)	*X*_2_ (deg/s)	*X*_3_(deg/s)	*Y*_4_ (m/s^2^)	*Y*_5_ (m/s^2^)	*Y*_6_ (m/s^2^)
**MAE**	**Vanilla LSTM**	0.318 (±0.13)	0.221 (±0.07)	0.225 (±0.07)	0.281 (±0.12)	0.369 (±0.10)	0.379 (±0.14)
	**Stacked LSTM**	0.288 (±0.15)	**0.176 (±0.10)**	0.185 (±0.11)	0.261 (±0.16)	**0.317 (±0.12)**	0.340 (±0.16)
	**Bi-LSTM**	0.314 (±0.13)	0.204 (±0.07)	0.212 (±0.08)	0.293 (±0.13)	0.363 (±0.10)	0.365 (±0.28)
	**ED-LSTM**	**0.276 (±0.14)**	**0.176 (±0.09)**	**0.184 (±0.09)**	**0.247 (±0.13)**	0.322 (±0.12)	**0.336 (±0.15)**
**MSE**	**Vanilla LSTM**	0.293 (±0.35)	0.112 (±0.12)	0.106 (±0.08)	0.232 (±0.26)	0.311 (±0.18)	0.348 (±0.26)
	**Stacked LSTM**	0.257 (±0.34)	**0.092 (±0.13)**	**0.092 (±0.13)**	0.237 (±0.32)	**0.277 (±0.21)**	0.314 (±0.28)
	**Bi-LSTM**	0.261 (±0.29)	0.112 (±0.13)	0.109 (±0.10)	0.256 (±0.33)	0.316 (±0.19)	0.334 (±0.24)
	**ED-LSTM**	**0.240 (±0.31)**	0.097 (±0.14)	**0.092 (±0.11)**	**0.202 (±0.25)**	**0.276 (±0.21)**	**0.299 (±0.25)**
**RMSE**	**Vanilla LSTM**	0.478 (±0.25)	0.304 (±0.14)	0.304 (±0.11)	0.425 (±0.22)	0.532 (±0.17)	0.549 (±0.22)
	**Stacked LSTM**	0.425 (±0.27)	0.250 (±0.17)	0.254 (±0.16)	0.396 (±0.28)	0.478 (±0.22)	0.497 (±0.25)
	**Bi-LSTM**	0.457 (±0.22)	0.296 (±0.15)	0.299 (±0.14)	0.439 (±0.25)	0.534 (±0.17)	0.538 (±0.21)
	**ED-LSTM**	**0.419 (±0.25)**	**0.257 (±0.17)**	**0.262 (±0.15)**	**0.374 (±0.24)**	**0.481 (±0.20)**	**0.490 (±0.24)**

Each of the predicted variables (i.e. *X*_1_, *X*_1_, …, *Y*_6_) was evaluated for the 4 trained LSTM architectures.

**Table 4:** Leave-one-out cross validation test error based on the NRMSE (%) at the PWS and 5km.h^-1^ combined.

Architecture	*X*_1_%	*X*_2_%	*X*_3_%	*Y*_4_%	*Y*_5_%	*Y*_6_%
**Vanilla LSTM**	10.71 (±5.25)	7.06 (±2.37)	7.13 (±2.39)	7.51 (±2.99)	10.14 (±3.39)	11.14 (±4.55)
**Stacked LSTM**	9.53 (±6.22)	5.742 (±3.40)	5.898 (±3.44)	6.933 (±4.17)	9.083 (±4.34)	9.882 (±4.96)
**Bi-LSTM**	6.983 (±5.0)	4.715 (±2.75)	4.807 (±3.23)	5.254 (±3.16)	7.643 (±3.76)	7.837 (±4.35)
**ED-LSTM**	**5.317 (±5.5)**	**2.823 (±3.33)**	**3.256 (±3.58)**	**2.960 (±3.77)**	**5.310 (±4.51)**	**5.287 (±5.02)**

Each of the predicted variables (i.e. *X*_1_, *X*_1_, …, *Y*_6_) was evaluated for the 4 trained LSTM architectures.

**Table 5:** Leave-one-out cross validation test evaluation based on the CC at the PWS and 5km.h^-1^ combined.

Architecture	*X*_1_	*X*_2_	*X*_3_	*Y*_4_	*Y*_5_	*Y*_6_
**Vanilla LSTM**	0.86 (±0.15)	0.94 (±0.05)	0.95 (±0.03)	0.88 (±0.11)	0.77 (±0.17)	0.76 (±0.18)
**Stacked LSTM**	0.87 (±0.16)	**0.95 (±0.06)**	0.95 (±0.04)	0.88 (±0.13)	0.77 (±0.20)	0.77 (±0.20)
**Bi-LSTM**	0.88 (±0.14)	**0.95 (±0.06)**	0.95 (±0.04)	0.88 (±0.13)	0.77 (±0.17)	0.77 (±0.18)
**ED-LSTM**	**0.89 (±0.14)**	**0.95 (±0.06)**	**0.96 (±0.04)**	**0.90 (±0.11)**	**0.80 (±0.18)**	**0.79 (±0.18)**

Each of the predicted variables (i.e. *X*_1_, *X*_1_, …, *Y*_6_) was evaluated for the 4 trained LSTM architectures.

## Discussion

The aim of this study was to investigate the capability of 4 LSTM architectures in predicting future lower limb trajectories of sagittal plane kinematic variables at an imposed speed (5k/h) and PWS. The predicted kinematic variables are the foot AV (X_1_), shank AV (X_2_), thigh AV (X_3_), foot LA (Y_4_), shank LA (Y_5_) and thigh LA (Y_6_). Prediction was performed using: (i) Vanilla LSTM, (ii) Stacked LSTM, (iii) Bi-LSTM and (iv) ED-LSTM. Results suggested that stacked and ED LSTM models are more reliable in predicting the future trajectory of the lower limb kinematics up to 0.1s (5 time-steps) (Fig 6). The ED-LSTM achieved the most accurate predicted kinematic trajectories among the other LSTM architectures (see Table 3). The prediction of future gait trajectory has the potential to expand the horizon of solving several problems in human movement science. In general, the LSTM models are designed to predict the future trajectories of any timeseries variable related to the human motion (e.g. knee angle). A known future gait trajectory adds a feedforward term to powered exoskeleton devices instead of predominantly relying on feedback sensors [1,27,29,69]. This would improve device performance by narrowing down the nonlinear kinematic differences between the user and the device and therefore avoid altering the user’s natural gait trajectories [70,71]. Prediction of future gait trajectory could substantially improve the design of prosthetics by adapting the device controlling parameters according to the user’s movement [72]. Additionally, a known 0.1s future gait trajectory falls in the range of slow and fast twitch motor units [73] and may allow a response time (10-120ms) for an elderly patient to adjust their gait and avoid an imminent risk of tripping or balance loss [73–77].

Four metrics were implemented to evaluate the LSTM prediction quality (Tables 3–5). Results have shown that LSTM predictions based on LA were worse than predictions based on AV in all models, possibly due to the double derivative generating a noisier signal (Fig 4). The foot AV (X_1_) predictions showed greater error (MAE 0.276–0.318 deg/s) throughout models compared to the shank (MAE 0.176–0.221 deg/s) and thigh (MAE 0.184–0.225 deg/s) AV, likely due to the grater variation of the foot trajectory throughout the gait cycle [40]. The NRMSE was used to facilitate the comparison between the LSTM models performance and to simplify the understanding of error rates for cross-disciplinary research. The Vanilla LSTM attained generalisation earlier (100 epochs) compared to all other models due to its simplicity and the fewer required parameters to train [57]. Albeit, it obtained higher error rates compared to the Stacked LSTM and the ED-LSTM (Fig 6 and Table 4). The Bi-LSTM have shown a competent prediction quality in this research (Table 4).

All models were good at predicting the signal patterns but were erroneous at obtaining the actual trajectories (see Fig 5). This indicates that prediction at PWS further challenges the prediction quality and it can be improved by training and testing the model on the same participant. The walking speed was found to be the most influential variable amongst sex, age and body max index on the ambulatory kinematic and kinetic profiles [78]. The changes of speed are known to have substantial impacts on the spatiotemporal as well as the kinematic and kinetic patterns of the gait cycle among different age groups [79,80]. Normal (comfortable or preferred) walking speed reported in the literature had averages ranging from 1.05m.s^-1^ to 1.43m.s^-1^ (cadence of 101 to 122 steps/min,) [81,82]. The imposed speed 5km.h^-1^ was found to be the general average PWS in previous studies [83–86] and it was adopted in this work to generalise the LSTM models to populations outside the recruited participants cohort. Prediction at the imposed walking speed of 5km.h^-1^ was found to be good in our previous work using the ED-LSTM [48] and in the literature using the PPCA [27]. The prediction at PWS however, allows the development of ML models that are better suited to individuals who might have different PWS which in return naturalise the human-machine (i.e. bionics) interface.

In our previous paper, we found that prediction of future kinematics trajectory (up to 0.06 s) was possible at an imposed speed (5km.h^-1^) using ED-LSTM. In this work we have expanded the prediction horizon up to 0.1s and investigated the other LSTM architectures to predict the kinematics trajectory at PWS and imposed speed (5km.h^-1^). The input (25 timesteps– 0.5s) and output (5 timesteps– 0.1s) sliding window sizes were designed as per the work by Zaroug *et al*. [48]. The combination of PWS and imposed speed 5km.h^-1^ timeseries data, widens the variability in the training data and further challenges the ML model to maintain a good prediction quality across different walking speeds. A ML model trained on PWS allows a generalised predictive model that could be fine tuned across different participants. The number of participants was increased for both genders (Male and female) to our previous work [48]. The foot segment was also added as the foot is the most distal segment of the human locomotor multisegment chain and plays an important part in maintaining balance, support and locomotion. Foot’s movement directly affects the lower extremities (i.e. ankle) dynamics and control [87] as well as the body’s Centre of Mass (COM) movement [88,89].

In contrast to the predicted trajectories evaluation technique by Tanghe et al. [27] (i.e. 3 selected time-steps), the performance evaluations of this work were calculated relative to the mean based on each of the predicted 75 time-steps (i.e. 1.5s). The ED-LSTM in this paper attained lower NRMSE range (2.83–5.31%) than the CNN implemented by Gholami et al. (6.5–11.1%) [40]. These results suggest that ED LSTM neural network is a more suitable model to capture features related to sequential time-series lower limb kinematic data [42,44]. The ED LSTM achieved the most accurate predictions in this paper (see Table 3). As demonstrated in the ED-LSTM architecture (see S1 Appendix) and in our previous paper [48], the internal learning process for the ED LSTM is unsupervised [90]. The ED LSTM obtains predictions based on a two learning phases. The encoder maps the input data (i.e. the input window) into a hidden layer and learns a compressed feature representation of the independent variables. While the decoder reconstructs the input data from the hidden layer to obtain the target dependent variables from the compressed feature representation. The optimiser (i.e. SGD) then minimises the reconstruction error which is the difference between the input and the reconstructed output [5]. This type of learning approach allowed the ED LSTM to obtain quality features from a given input of kinematic trajectories [91]. The unsupervised feature learning paradigm (i.e. encoder) and the reconstruction of time series information (i.e. decoder) has made the ED LSTM a good architecture for high level deep features extraction and for generative models [90,92,93]. The complexity and the power of the ED LSTM could be extended to other learning techniques such as the unsupervised greedy layer-wise pre-training referred to as pretraining [57]. The pretraining involves the training of a shallow layer and sequentially adding up and refitting a new hidden layer to learn inputs from the existing previous layer (i.e. shallow layer) while keeping fixed the learned weights and biases of the previous layer [94]. The pretraining structure opened up the opportunity to train very deep stacked ED LSTM with less possibility of overfitting (because the training is performed in layer-wise) and a lower generalisation error [95].

This work was limited to 15 young healthy participants. There exists a proportional relationship between the human age and their walking speeds. The walking speed was found to be slightly decreasing each year among healthy male and female populations [96]. Future work is needed to test the models’ reliability on predicting slower speeds than that tested and accommodate predictions related to elderly population who may walk slower. Patients with balance issues or fall history should be recruited to further understand the potential application of this work for falls prevention [74,75,97,98]. Finally, a more complex model such as the hybrid models (i.e. ConvLSTM) or a different learning technique such as the greedy-layer wise pre-training [95] may help expand the prediction horizon while maintaining the prediction quality.

## Conclusions

In this work we developed and compared 4 LSTM architectures for the prediction of future lower limb kinematics (i.e. foot AV, shank AV, thigh AV, foot LA, shank LA and thigh LA). Results suggested that future lower limb kinematics while walking at PWS and at 5km.h^-1^ can be well predicted up to 0.1s with ED-LSTM and Stacked LSTM. These findings highlight the potential of LSTM neural networks to predict the future trajectories of the human movements. This could have application in exoskeleton control systems or falls prevention. Further work is needed to understand the model’s robustness under different walking conditions and in participants with a pathological gait. Future directions would incorporate other LSTM baseline varients such as the Gated Recurrent Unit (GRU) and the LSTM with attention or self-attention.

## Supporting information

S1 Appendix Implemented LSTM architectures. (PDF)

S1 Data (CSV)

## References

[1] Anam K, Al-Jumaily AA. Active exoskeleton control systems: State of the art. Procedia Engineering. 2012;41:988–94.

[2] Sawicki GS, Beck ON, Kang I, Young AJ. The exoskeleton expansion: improving walking and running economy. Journal of NeuroEngineering and Rehabilitation. 2020;17(1):1–9. 32075669 10.1186/s12984-020-00663-9 PMC7029455

[3] Ahn S, Kim J, Koo B, Kim Y. Evaluation of inertial sensor-based pre-impact fall detection algorithms using public dataset. Sensors. 2019;19(4):774. doi: 10.3390/s19040774 30781886 PMC6412321

[4] Hori K, Mao Y, Ono Y, Ora H, Hirobe Y, Sawada H, et al. Inertial measurement unit-based estimation of foot trajectory for clinical gait analysis. Frontiers in Physiology. 2019;10. doi: 10.3389/fphys.2019.01530 31998138 PMC6966410

[5] Lai DT, Begg RK, Palaniswami M. Computational intelligence in gait research: a perspective on current applications and future challenges. IEEE Transactions on Information Technology in Biomedicine. 2009;13(5):687–702. doi: 10.1109/TITB.2009.2022913 19447724

[6] Srinivasan S, Raptis I, Westervelt ER. Low-dimensional sagittal plane model of normal human walking. Journal of biomechanical engineering. 2008;130(5). doi: 10.1115/1.2970058 19045524

[7] Zajac FE, Winters JM. Modeling musculoskeletal movement systems: joint and body segmental dynamics, musculoskeletal actuation, and neuromuscular control. Multiple muscle systems: Springer; 1990. p. 121–48.

[8] Yamaguchi GT. Performing whole-body simulations of gait with 3-D, dynamic musculoskeletal models. Multiple Muscle Systems: Springer; 1990. p. 663–79.

[9] Marshall R, Wood G, Jennings L. Performance objectives in human movement: A review and application to the stance phase of normal walking. Human Movement Science. 1989;8(6):571–94.

[10] Pandy MG. Computer modeling and simulation of human movement. Annual review of biomedical engineering. 2001;3(1):245–73. doi: 10.1146/annurev.bioeng.3.1.245 11447064

[11] Chevallereau C, Aoustin Y. Optimal reference trajectories for walking and running of a biped robot. Robotica. 2001;19(5):557–69.

[12] Ren L, Jones RK, Howard D. Predictive modelling of human walking over a complete gait cycle. Journal of biomechanics. 2007;40(7):1567–74. doi: 10.1016/j.jbiomech.2006.07.017 17070531

[13] Kuo AD. A simple model of bipedal walking predicts the preferred speed–step length relationship. J Biomech Eng. 2001;123(3):264–9. doi: 10.1115/1.1372322 11476370

[14] Kuo AD, Donelan JM, Ruina A. Energetic consequences of walking like an inverted pendulum: step-to-step transitions. Exercise and sport sciences reviews. 2005;33(2):88–97. doi: 10.1097/00003677-200504000-00006 15821430

[15] Vargas-Valencia LS, Elias A, Rocon E, Bastos-Filho T, Frizera A. An IMU-to-body alignment method applied to human gait analysis. Sensors. 2016;16(12):2090. doi: 10.3390/s16122090 27973406 PMC5191070

[16] Sabatini AM. Estimating three-dimensional orientation of human body parts by inertial/magnetic sensing. Sensors. 2011;11(2):1489–525. doi: 10.3390/s110201489 22319365 PMC3274035

[17] Halilaj E, Rajagopal A, Fiterau M, Hicks JL, Hastie TJ, Delp SL. Machine learning in human movement biomechanics: best practices, common pitfalls, and new opportunities. Journal of biomechanics. 2018;81:1–11. doi: 10.1016/j.jbiomech.2018.09.009 30279002 PMC6879187

[18] Phinyomark A, Petri G, Ibáñez-Marcelo E, Osis ST, Ferber R. Analysis of big data in gait biomechanics: Current trends and future directions. Journal of medical and biological engineering. 2018;38(2):244–60. doi: 10.1007/s40846-017-0297-2 29670502 PMC5897457

[19] Mitchell TM. Machine learning. McGraw-hill New York; 1997.

[20] Begg R, Palaniswami M. Computational intelligence for movement sciences: neural networks and other emerging techniques: IGI Global; 2006.

[21] Lai DT, Levinger P, Begg RK, Gilleard WL, Palaniswami M. Automatic recognition of gait patterns exhibiting patellofemoral pain syndrome using a support vector machine approach. IEEE Transactions on Information Technology in Biomedicine. 2009;13(5):810–7. doi: 10.1109/TITB.2009.2022927 19447723

[22] Begg RK, Palaniswami M, Owen B. Support vector machines for automated gait classification. IEEE Transactions on Biomedical Engineering. 2005;52(5):828–38. doi: 10.1109/TBME.2005.845241 15887532

[23] Begg R, Kamruzzaman J. A machine learning approach for automated recognition of movement patterns using basic, kinetic and kinematic gait data. Journal of biomechanics. 2005;38(3):401–8. doi: 10.1016/j.jbiomech.2004.05.002 15652537

[24] Kamruzzaman J, Begg RK. Support vector machines and other pattern recognition approaches to the diagnosis of cerebral palsy gait. IEEE Transactions on Biomedical Engineering. 2006;53(12):2479–90. 17153205 10.1109/TBME.2006.883697

[25] Chong E, Park FC. Movement prediction for a lower limb exoskeleton using a conditional restricted Boltzmann machine. Robotica. 2017;35(11):2177–200.

[26] Ding Y, Kim M, Kuindersma S, Walsh CJ. Human-in-the-loop optimization of hip assistance with a soft exosuit during walking. Sci Robot. 2018;3(15):eaar5438. doi: 10.1126/scirobotics.aar5438 33141683

[27] Tanghe K, De Groote F, Lefeber D, De Schutter J, Aertbeliën E. Gait Trajectory and Event Prediction from State Estimation for Exoskeletons during Gait. IEEE Transactions on Neural Systems and Rehabilitation Engineering. 2019. doi: 10.1109/TNSRE.2019.2950309 31675336

[28] Begg R, Lai DT, Palaniswami M. Computational intelligence in biomedical engineering: CRC Press; 2007.

[29] Zaroug A, Proud JK, Lai DT, Mudie K, Billing D, Begg R. Overview of Computational Intelligence (CI) Techniques for Powered Exoskeletons. Computational Intelligence in Sensor Networks: Springer; 2019. p. 353–83.

[30] LeCun Y, Bengio Y, Hinton G. Deep learning. nature. 2015;521(7553):436. doi: 10.1038/nature14539 26017442

[31] Murad A, Pyun J-Y. Deep recurrent neural networks for human activity recognition. Sensors. 2017;17(11):2556. doi: 10.3390/s17112556 29113103 PMC5712979

[32] Han B-K, Ryu J-K, Kim S-C. Context-Aware Winter Sports Based on Multivariate Sequence Learning. Sensors. 2019;19(15):3296. doi: 10.3390/s19153296 31357531 PMC6696288

[33] Fernandez-Lopez P, Liu-Jimenez J, Kiyokawa K, Wu Y, Sanchez-Reillo R. Recurrent neural network for inertial gait user recognition in smartphones. Sensors. 2019;19(18):4054. doi: 10.3390/s19184054 31546976 PMC6767850

[34] Horst F, Lapuschkin S, Samek W, Müller K-R, Schöllhorn WI. Explaining the unique nature of individual gait patterns with deep learning. Scientific reports. 2019;9(1):2391. doi: 10.1038/s41598-019-38748-8 30787319 PMC6382912

[35] Jung J-Y, Heo W, Yang H, Park H. A Neural Network-Based Gait Phase Classification Method Using Sensors Equipped on Lower Limb Exoskeleton Robots. Sensors. 2015;15(11):27738. doi: 10.3390/s151127738 26528986 PMC4701252

[36] Trigili E, Grazi L, Crea S, Accogli A, Carpaneto J, Micera S, et al. Detection of movement onset using EMG signals for upper-limb exoskeletons in reaching tasks. Journal of neuroengineering and rehabilitation. 2019;16(1):45. doi: 10.1186/s12984-019-0512-1 30922326 PMC6440169

[37] Moon D-H, Kim D, Hong Y-D. Development of a Single Leg Knee Exoskeleton and Sensing Knee Center of Rotation Change for Intention Detection. Sensors. 2019;19(18):3960. doi: 10.3390/s19183960 31540298 PMC6767670

[38] Islam M, Hsiao-Wecksler ET. Detection of gait modes using an artificial neural network during walking with a powered ankle-foot orthosis. Journal of Biophysics. 2016;2016. doi: 10.1155/2016/7984157 28070188 PMC5187599

[39] Findlow A, Goulermas J, Nester C, Howard D, Kenney L. Predicting lower limb joint kinematics using wearable motion sensors. Gait & posture. 2008;28(1):120–6. doi: 10.1016/j.gaitpost.2007.11.001 18093834

[40] Gholami M, Napier C, Menon C. Estimating Lower Extremity Running Gait Kinematics with a Single Accelerometer: A Deep Learning Approach. Sensors. 2020;20(10):2939. doi: 10.3390/s20102939 32455927 PMC7287664

[41] Graves A. Generating sequences with recurrent neural networks. arXiv preprint arXiv:13080850. 2013.

[42] Graves A. Supervised sequence labelling. Supervised sequence labelling with recurrent neural networks: Springer; 2012. p. 5–13.

[43] Su B, Gutierrez-Farewik EM. Gait Trajectory and Gait Phase Prediction Based on an LSTM Network. Sensors. 2020;20(24):7127. 10.3390/s20247127 PMC7764336 33322673

[44] Zhao A, Qi L, Dong J, Yu H. Dual channel LSTM based multi-feature extraction in gait for diagnosis of Neurodegenerative diseases. Knowledge-Based Systems. 2018;145:91–7.

[45] Kidziński Ł, Delp S, Schwartz M. Automatic real-time gait event detection in children using deep neural networks. PloS one. 2019;14(1):e0211466. doi: 10.1371/journal.pone.0211466 30703141 PMC6354999

[46] Tan HX, Aung NN, Tian J, Chua MCH, Yang YO. Time series classification using a modified LSTM approach from accelerometer-based data: A comparative study for gait cycle detection. Gait & posture. 2019;74:128–34. doi: 10.1016/j.gaitpost.2019.09.007 31518859

[47] Nait Aicha A, Englebienne G, van Schooten K, Pijnappels M, Kröse B. Deep learning to predict falls in older adults based on daily-life trunk accelerometry. Sensors. 2018;18(5):1654. doi: 10.3390/s18051654 29786659 PMC5981199

[48] Zaroug A, Lai DT, Mudie K, Begg R. Lower Limb Kinematics Trajectory Prediction Using Long Short-Term Memory Neural Networks. Frontiers in Bioengineering and Biotechnology. 2020;8. doi: 10.3389/fbioe.2020.00362 32457881 PMC7227385

[49] Ichinosawa Y, Shimizu S, Takemura N, Taira K, Hamakawa M, Nakachi Y, et al. Gait speed and balance function strongly determine the ability to walk independently without using a wheelchair in a facility setting for stroke patients. The Kitasato medical journal. 2018;48(1):16–25.

[50] Alexander EJ, Andriacchi TP. Correcting for deformation in skin-based marker systems. Journal of biomechanics. 2001;34(3):355–61. doi: 10.1016/s0021-9290(00)00192-5 11182127

[51] Cappozzo A, Catani F, Della Croce U, Leardini A. Position and orientation in space of bones during movement: anatomical frame definition and determination. Clinical biomechanics. 1995;10(4):171–8. doi: 10.1016/0268-0033(95)91394-t 11415549

[52] Dyrby CO, Andriacchi TP. Secondary motions of the knee during weight bearing and non-weight bearing activities. Journal of Orthopaedic Research. 2004;22(4):794–800. doi: 10.1016/j.orthres.2003.11.003 15183436

[53] Garofolini A. Exploring adaptability in long-distance runners: effect of foot strike pattern on lower limb neuro-muscular-skeletal capacity: Victoria University; 2019.

[54] Butterworth S. On the theory of filter amplifiers. Wireless Engineer. 1930;7(6):536–41.

[55] Davis PJ. Interpolation and approximation: Courier Corporation; 1975.

[56] Soutas-Little RW. Motion analysis and biomechanics. J Rehabil Res Dev. 1998:49–68.

[57] Sagheer A, Kotb M. Unsupervised pre-training of a Deep LStM-based Stacked Autoencoder for Multivariate time Series forecasting problems. Scientific Reports. 2019;9(1):1–16. 31836728 10.1038/s41598-019-55320-6 PMC6911101

[58] Géron A. Hands-on machine learning with Scikit-Learn, Keras, and TensorFlow: Concepts, tools, and techniques to build intelligent systems: O’Reilly Media; 2019.

[59] Francois C. Deep learning with Python. Manning Publications Company; 2017.

[60] Sra S, Nowozin S, Wright SJ. Optimization for machine learning: Mit Press; 2012.

[61] Bottou L. Large-scale machine learning with stochastic gradient descent. Proceedings of COMPSTAT’2010: Springer; 2010. p. 177–86.

[62] Bottou L. Stochastic gradient descent tricks. Neural networks: Tricks of the trade: Springer; 2012. p. 421–36.

[63] Rumelhart DE, Hinton GE, Williams RJ. Learning representations by back-propagating errors. nature. 1986;323(6088):533–6.

[64] Sutskever I, Martens J, Dahl G, Hinton G, editors. On the importance of initialization and momentum in deep learning. International conference on machine learning; 2013.

[65] Graves A, Schmidhuber J. Framewise phoneme classification with bidirectional LSTM and other neural network architectures. Neural networks. 2005;18(5–6):602–10. doi: 10.1016/j.neunet.2005.06.042 16112549

[66] Banos O, Galvez J-M, Damas M, Pomares H, Rojas I. Window size impact in human activity recognition. Sensors. 2014;14(4):6474–99. doi: 10.3390/s140406474 24721766 PMC4029702

[67] Schöllhorn W, Nigg B, Stefanyshyn D, Liu W. Identification of individual walking patterns using time discrete and time continuous data sets. Gait & Posture. 2002;15(2):180–6. doi: 10.1016/s0966-6362(01)00193-x 11869912

[68] Horst F, Mildner M, Schöllhorn W. One-year persistence of individual gait patterns identified in a follow-up study–A call for individualised diagnose and therapy. Gait & posture. 2017;58:476–80. 28926814 10.1016/j.gaitpost.2017.09.003

[69] Proud JK, Lai DT, Mudie KL, Carstairs GL, Billing DC, Garofolini A, et al. Exoskeleton Application to Military Manual Handling Tasks. Human Factors. 2020:0018720820957467. doi: 10.1177/0018720820957467 33203237

[70] Torricelli D, Cortés C, Lete N, Bertelsen Simonetti Á, Gonzalez-Vargas J, del-Ama AJ, et al. A subject-specific kinematic model to predict human motion in exoskeleton-assisted gait. Frontiers in neurorobotics. 2018;12:18. doi: 10.3389/fnbot.2018.00018 29755336 PMC5934493

[71] Rupal BS, Rafique S, Singla A, Singla E, Isaksson M, Virk GS. Lower-limb exoskeletons: Research trends and regulatory guidelines in medical and non-medical applications. International Journal of Advanced Robotic Systems. 2017;14(6):1729881417743554.

[72] Shafiul Hasan S, Siddiquee MR, Atri R, Ramon R, Marquez JS, Bai O. Prediction of gait intention from pre-movement EEG signals: a feasibility study. Journal of NeuroEngineering and Rehabilitation. 2020;17:1–16. 32299460 10.1186/s12984-020-00675-5 PMC7164221

[73] Winter DA. Biomechanics and motor control of human movement: John Wiley & Sons; 2009.

[74] Barrett R, Mills P, Begg R. A systematic review of the effect of ageing and falls history on minimum foot clearance characteristics during level walking. Gait & posture. 2010;32(4):429–35. doi: 10.1016/j.gaitpost.2010.07.010 20692163

[75] Lai DT, Taylor SB, Begg RK. Prediction of foot clearance parameters as a precursor to forecasting the risk of tripping and falling. Human movement science. 2012;31(2):271–83. doi: 10.1016/j.humov.2010.07.009 21035220

[76] Begg RK, Rahman SM. A method for the reconstruction of ground reaction force-time characteristics during gait from force platform recordings of simultaneous foot falls. IEEE transactions on biomedical engineering. 2000;47(4):547–51. doi: 10.1109/10.828154 10763300

[77] Khandoker AH, Lai D, Begg RK, Palaniswami M, editors. A wavelet-based approach for screening falls risk in the elderly using support vector machines. 2006 Fourth International Conference on Intelligent Sensing and Information Processing; 2006: IEEE.

[78] Chehab E, Andriacchi T, Favre J. Speed, age, sex, and body mass index provide a rigorous basis for comparing the kinematic and kinetic profiles of the lower extremity during walking. Journal of biomechanics. 2017;58:11–20. doi: 10.1016/j.jbiomech.2017.04.014 28501342

[79] Fukuchi CA, Fukuchi RK, Duarte M. Effects of walking speed on gait biomechanics in healthy participants: a systematic review and meta-analysis. Systematic reviews. 2019;8(1):153. doi: 10.1186/s13643-019-1063-z 31248456 PMC6595586

[80] Grant J, Chester V. The effects of walking speed on adult multi-segment foot kinematics. Journal of Bioengineering & Biomedical Science. 2015;5(2):181–3.

[81] Kwon JW, Son SM, Lee NK. Changes of kinematic parameters of lower extremities with gait speed: a 3D motion analysis study. Journal of Physical Therapy Science. 2015;27(2):477–9. doi: 10.1589/jpts.27.477 25729195 PMC4339165

[82] Winter DA. Biomechanics and motor control of human gait: normal, elderly and pathological 1991.

[83] Browning RC, Baker EA, Herron JA, Kram R. Effects of obesity and sex on the energetic cost and preferred speed of walking. Journal of applied physiology. 2006;100(2):390–8. doi: 10.1152/japplphysiol.00767.2005 16210434

[84] Mohler BJ, Thompson WB, Creem-Regehr SH, Pick HL, Warren WH. Visual flow influences gait transition speed and preferred walking speed. Experimental brain research. 2007;181(2):221–8. doi: 10.1007/s00221-007-0917-0 17372727

[85] Waters RL, Lunsford BR, Perry J, Byrd R. Energy-speed relationship of walking: standard tables. Journal of Orthopaedic Research. 1988;6(2):215–22. doi: 10.1002/jor.1100060208 3343627

[86] Mohamed O, Appling H. Clinical Assessment of Gait. 2020. In: Orthotics and Prosthetics in Rehabilitation (Fourth Edition) [Internet]. Elsevier. 4. [102–43]. (http://www.sciencedirect.com/science/article/pii/B9780323609135000052).

[87] Garofolini A, Taylor S, Mclaughlin P, Mickle KJ, Frigo CA. Ankle Joint Dynamic Stiffness in Long-Distance Runners: Effect of Foot Strike and Shoes Features. Applied Sciences. 2019;9(19):4100.

[88] Chan CW, Rudins A, editors. Foot biomechanics during walking and running. Mayo Clinic Proceedings; 1994: Elsevier. 10.1016/s0025-6196(12)61642-5 8170197

[89] Mun K-R, Song G, Chun S, Kim J. Gait estimation from anatomical foot parameters measured by a foot feature measurement system using a deep neural network model. Scientific reports. 2018;8(1):1–10. 29959364 10.1038/s41598-018-28222-2 PMC6026202

[90] Srivastava N, Mansimov E, Salakhudinov R, editors. Unsupervised learning of video representations using lstms. International conference on machine learning; 2015.

[91] Zabalza J, Ren J, Zheng J, Zhao H, Qing C, Yang Z, et al. Novel segmented stacked autoencoder for effective dimensionality reduction and feature extraction in hyperspectral imaging. Neurocomputing. 2016;185:1–10.

[92] Blaschke T, Olivecrona M, Engkvist O, Bajorath J, Chen H. Application of generative autoencoder in de novo molecular design. Molecular informatics. 2018;37(1–2):1700123. doi: 10.1002/minf.201700123 29235269 PMC5836887

[93] Bao W, Yue J, Rao Y. A deep learning framework for financial time series using stacked autoencoders and long-short term memory. PloS one. 2017;12(7):e0180944. doi: 10.1371/journal.pone.0180944 28708865 PMC5510866

[94] Goodfellow I, Bengio Y, Courville A, Bengio Y. Deep learning: MIT press Cambridge; 2016.

[95] Bengio Y, Lamblin P, Popovici D, Larochelle H. Greedy layer-wise training of deep networks. Advances in neural information processing systems. 2006;19:153–60.

[96] Schimpl M, Moore C, Lederer C, Neuhaus A, Sambrook J, Danesh J, et al. Association between walking speed and age in healthy, free-living individuals using mobile accelerometry—a cross-sectional study. PloS one. 2011;6(8):e23299. doi: 10.1371/journal.pone.0023299 21853107 PMC3154324

[97] Levinger P, Nagano H, Downie C, Hayes A, Sanders KM, Cicuttini F, et al. Biomechanical balance response during induced falls under dual task conditions in people with knee osteoarthritis. Gait & posture. 2016;48:106–12. doi: 10.1016/j.gaitpost.2016.04.031 27239773

[98] Nagano H, Said CM, James L, Begg RK. Feasibility of Using Foot–Ground Clearance Biofeedback Training in Treadmill Walking for Post-Stroke Gait Rehabilitation. Brain Sciences. 2020;10(12):978. doi: 10.3390/brainsci10120978 33322082 PMC7764443

