# Supplementary material for: Prediction of gait trajectories based on the Long Short Term Memory neural networks
Source: PLoS One. 2021 Aug 5;16(8):e0255597. doi: 10.1371/journal.pone.0255597 (PMC8341582; doi:10.1371/journal.pone.0255597)
Supplement: S1 Appendix — (PDF) [file pone.0255597.s001.pdf]

S1 Appendix

Prediction of gait trajectories based on the Long Short Term Memory neural networks

Abdelrahman Zaroug[1¶*], Alessandro Garofolini[1&] , Daniel T.H. Lai[1,2&], Kurt Mudie[3&], Rezaul Begg[1&]

[1]Institute for Health and Sport, Victoria University, Melbourne, Victoria, Australia

[2]College of Engineering and Science, Victoria University, Melbourne, Victoria, Australia

[3]Defence Science and Technology Group, Melbourne, Victoria, Australia

*Corresponding author
E-mail: abdelrahman.zaroug@live.vu.edu.au (AZ)

[¶] These authors contributed equally to this work

[&] These authors contributed equally to this work

Long Short-Term Memory (LSTM) neural networks

Standard RNNs can be trained to learn sequences one step at a time and predict what comes next (Graves, 2013). However, as the network becomes deeper and the error signal (i.e. BPTT) flows back in time to update the first units in the network, the error gradient tends to either attenuate or amplify exponentially known as, respectively, the vanishing and the exploding gradient (Hochreiter, Bengio, Frasconi, & Schmidhuber, 2001; Pascanu, Mikolov, & Bengio, 2013; Rumelhart, Hinton, & Williams, 1985). As a result, the gradient is prevented from carrying information across the network and the model would become incapable of capturing temporally distant events (Graves, 2013; Graves & Schmidhuber, 2005). In response to address some of the RNN shortcomings, LSTM was proposed by Hochreiter and Schmidhuber in 1997 (Hochreiter & Schmidhuber, 1997).

LSTM is an alternative RNN architecture designed to better store information than standard RNN due to its cell state vectors which retain information over a longer period by using an explicit gating mechanism (Graves, 2012; Hochreiter & Schmidhuber, 1997; Karpathy, Johnson, & Fei-Fei, 2015). The cell state derivative is the term used to prevent the LSTM gradients from vanishing. The LSTM however, doesn't explicitly address the exploding gradients problem (Pascanu et al., 2013). The exploding gradients occurs during the training process when there is a large increase in the gradients norm caused by substantial increase in the long term components than the short term (Bengio, Simard, & Frasconi, 1994). Gradient norm clipping was the mechanism implemented in this paper to curb exploding gradients (Pascanu et al., 2013). It works by rescaling the gradient norm whenever it exceeds a specific threshold, which forces the optimiser (i.e. SGD) to jump across different local minima and therefore avoids exploding gradients.

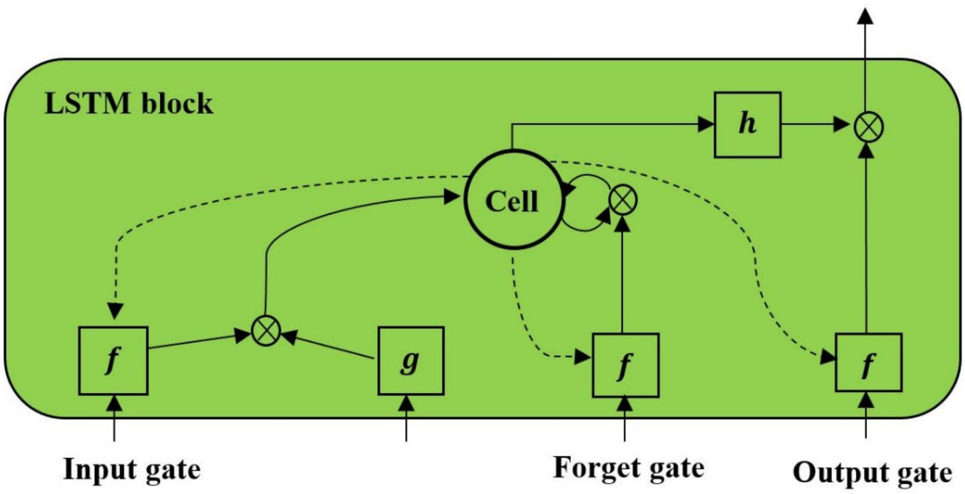

Fig S1: Standard LSTM unit. Each gate is a nonlinear summation unit that collects activations from inside and outside the block. The gates have also control over the memory cell activations using multiplication operation (the small crossed circles).

Each LSTM cell has an input gate, forget gate and output gate. The input gate dictates the information used to update the memory state and the forget gate decides which information to discard or remove from the cell. The final gate specifies the information to output based on the cell input and memory. All gates are designed such that information is exchanged from inside and outside the block (Fig S1). Furthermore, each memory block contains three peephole-weighted connections (dotted lines in Fig S1) which are the input weight $w_{c\iota}$, the output weight $w_{c\omega}$ and the memory state $w_{c\phi}$. The functions f, g and h are usually tanh or logistic sigmoid (0 = gate closed and 1= gate open) activation functions (Graves, 2012). Below are the network equations (Graves, 2012) that govern the LSTM architecture used:

Input gates:

$$a_\iota^t = \sum_{i=1}^{I} w_{i\iota} x_i^t + \sum_{h=1}^{H} w_{h\iota} b_h^{t-1} + \sum_{c=1}^{C} w_{c\iota} s_c^{t-1} \qquad (1)$$

$$b_\iota^t = f(a_\iota^t) \qquad (2)$$

Forget gates:

$$a_{\phi}^{t} = \sum_{i=1}^{I} w_{i\phi} x_{i}^{t} + \sum_{h=1}^{H} w_{h\phi} b_{h}^{t-1} + \sum_{c=1}^{C} w_{c\phi} s_{c}^{t-1} \tag{3}$$

$$b_{\phi}^{t} = f(a_{\phi}^{t}) \tag{4}$$

Cells:

$$a_{c}^{t} = \sum_{i=1}^{I} w_{ic} x_{i}^{t} + \sum_{h=1}^{H} w_{hc} b_{h}^{t-1} \tag{5}$$

$$s_{c}^{t} = b_{\phi}^{t} s_{c}^{t-1} + b_{\iota}^{t} g(a_{c}^{t}) \tag{6}$$

Output gates:

$$a_{c}^{t} = \sum_{i=1}^{I} w_{iw} x_{i}^{t} + \sum_{h=1}^{H} w_{hw} b_{h}^{t-1} + \sum_{c=1}^{C} w_{cw} s_{c}^{t} \tag{7}$$

$$b_{w}^{t} = f(a_{w}^{t}) \tag{8}$$

Cell outputs:

$$b_{c}^{t} = b_{w}^{t} h(s_{c}^{t}) \tag{9}$$

Where, w_{ij} is the weight of the connection from unit i to unit j, a_j^t is the network input to unit j at time t, b_j^t is the activation of unit j at time t, ι, ϕ, ω are respectively stands for the input gate, forget gate, and output gate, C is the memory cell, $w_{c\iota}, w_{c\phi}, w_{c\omega}$ are peephole weights, s_c^t is the state of cell C at time t, f is the input, output and forget gates activation function, g *and* h are the cell input and output activations, I is the number of inputs, H is the number of cells in the hidden layer and index h is the cell outputs from other blocks in the hidden layer. Bias was neglected for simplicity.

Vanilla LSTM neural network

The vanilla LSTM neural network is the simplest and most commonly used LSTM architecture in the literature (Greff, Srivastava, Koutník, Steunebrink, & Schmidhuber, 2016; Wu, Yuan, Dong, Lin, & Liu, 2018). It is the architecture defined in the original LSTM paper (Hochreiter & Schmidhuber, 1997). It consists of single interconnected LSTM hidden layer and a final dense layer for outputting predictions (see Fig 5). In this work, there were 1024 LSTM units.

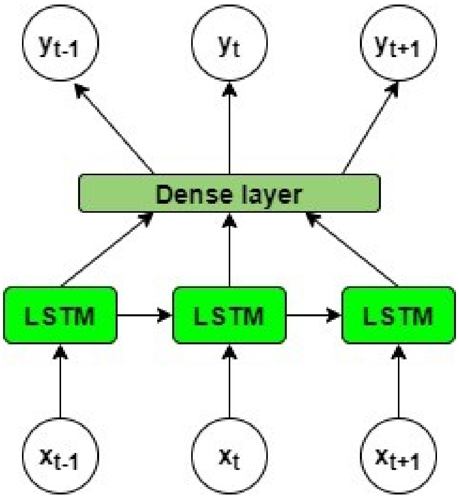

Fig S2: Vanilla LSTM neural networks (Wu et al., 2018).

Stacked LSTM neural network

It is widely understood that the generalisation of the LSTM model is a function of how deep the network is (Greff et al., 2016; Hermans & Schrauwen, 2013; Sagheer & Kotb, 2019a). A deeper neural network that consists of multiple LSTM hidden layers is known to have higher learning capacity (Hermans & Schrauwen, 2013). The stacked LSTM architecture as shown in Fig S3 consists of multiple LSTM hidden layers and a final dense layer for outputting predictions (Graves, 2013). In this work, there were 5 LSTM hidden layers each with 256 units.

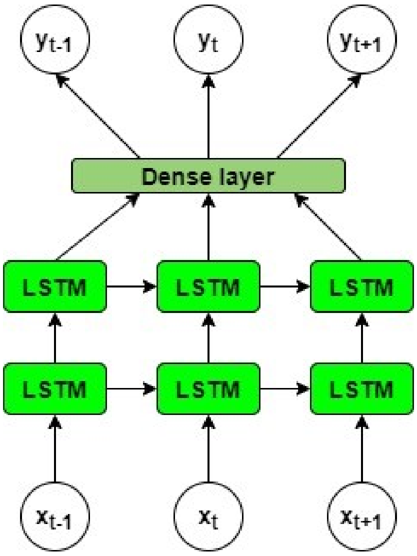

Fig S3: Stacked LSTM neural networks (Graves, 2013).

Bidirectional LSTM (Bi-LSTM) neural network

Bi-LSTM was introduced as an extension to RNN (Schuster & Paliwal, 1997). Given the entire input data is available, the focus of the bidirectional LSTM neural networks is to maximise modelling of the input sequence by processing the data in forward and backward directions. The architecture works by duplicating the hidden layer side-by-side (see Fig S4) and process the input sequence as-is to the original hidden layer (forward layer) and as a

reversed copy to the duplicate layer (backward layer). The states from the forward layer are not connected to the backward layer, however, both layers are connected to the output layer (Graves & Schmidhuber, 2005; Schuster & Paliwal, 1997). The means by which Bi-LSTM models the input sequence in this manner is the fact that there is a scientific evidence that humans may use sounds, or words that only make sense at a future context (Graves & Schmidhuber, 2005). In this work, there was a single Bi-LSTM with 1024 LSTM units and a Batch Normalisation (BN) applied to the inputs. The aim of the BN is to expedite the neural network training by stabilising the distributions (mean and variance) of the input layer (Ioffe & Szegedy, 2015). The technique was initially deemed to be successful because it reduces the Internal Covariate Shift (ICS), however, Santurkar *et al*. suggested that BN reparametrises the optimisation problem by smoothening the loss function landscape (Santurkar, Tsipras, Ilyas, & Madry, 2018). Therefore the optimisation problem become easier to solve (especially in the case of sharp minima or flat regions (Li, Xu, Taylor, Studer, & Goldstein, 2018)) and the gradients become reliable and predictive. BN was added to Bi-LSTM because the convergence to local minima was found slower compared to other LSTM models (Siami-Namini, Tavakoli, & Namin, 2019).

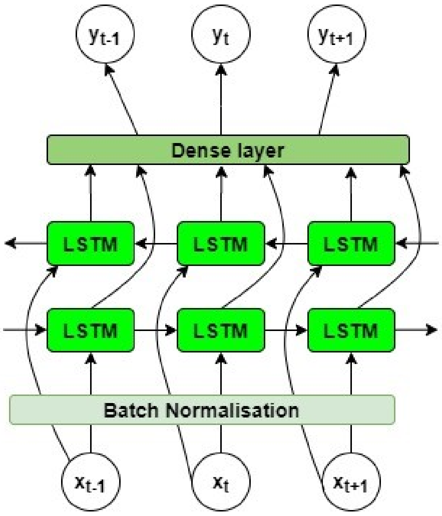

Fig S4: Bidirectional LSTM neural networks with a BN input layer (Schuster & Paliwal, 1997).

LSTM autoencoder (ED-LSTM) neural network

Proposed by Srivastava *et al*,. 2015, the LSTM autoencoder (see Fig S5) is an unsupervised learning method that seeks to learn from a fixed length input sequence representation (Srivastava, Mansimov, & Salakhudinov, 2015). Although it is normally trained with supervised learning, the LSTM autoencoder recreates the input sequence into a fixed length feature vector. It is composed of two main layers, the encoder layer and the decoder layer. At first, the encoder encodes the variable length input sequence into a fixed length feature vector that represents the input attributes. Then the decoder layer decodes the fixed length feature vector into a variable length output sequence. The general LSTM autoencoder model implemented in our last paper (Zaroug et al., 2020) was kept unchanged, except that the number of units was increased to 1024 per-side (encoder and decoder).

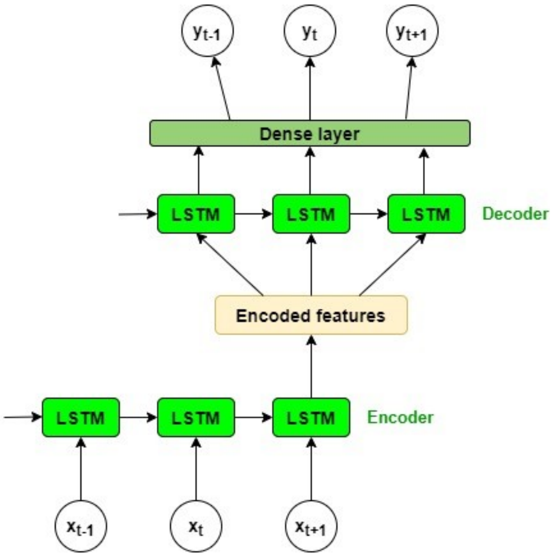

Fig S5: LSTM autoencoder architecture (Sagheer & Kotb, 2019b; Zaroug et al., 2020).

REFERENCES

Bengio, Y., Simard, P., & Frasconi, P. (1994). Learning long-term dependencies with gradient descent is difficult. *IEEE transactions on neural networks, 5*(2), 157-166.

Bottou, L. (2010). Large-scale machine learning with stochastic gradient descent. In *Proceedings of COMPSTAT'2010* (pp. 177-186): Springer.

Bottou, L. (2012). Stochastic gradient descent tricks. In *Neural networks: Tricks of the trade* (pp. 421-436): Springer.

Francois, C. (2017). Deep learning with Python. In: Manning Publications Company.

Géron, A. (2019). *Hands-on machine learning with Scikit-Learn, Keras, and TensorFlow: Concepts, tools, and techniques to build intelligent systems*: O'Reilly Media.

Graves, A. (2012). Supervised sequence labelling. In *Supervised sequence labelling with recurrent neural networks* (pp. 5-13): Springer.

Graves, A. (2013). Generating sequences with recurrent neural networks. *arXiv preprint arXiv:1308.0850*.

Graves, A., & Schmidhuber, J. (2005). Framewise phoneme classification with bidirectional LSTM and other neural network architectures. *Neural networks, 18*(5-6), 602-610.

Greff, K., Srivastava, R. K., Koutník, J., Steunebrink, B. R., & Schmidhuber, J. (2016). LSTM: A search space odyssey. *IEEE transactions on neural networks and learning systems, 28*(10), 2222-2232.

Hermans, M., & Schrauwen, B. (2013). *Training and analysing deep recurrent neural networks.* Paper presented at the Advances in neural information processing systems.

Hochreiter, S., Bengio, Y., Frasconi, P., & Schmidhuber, J. (2001). Gradient flow in recurrent nets: the difficulty of learning long-term dependencies. In: A field guide to dynamical recurrent neural networks. IEEE Press.

Hochreiter, S., & Schmidhuber, J. (1997). Long short-term memory. *Neural computation, 9*(8), 1735-1780.

Ioffe, S., & Szegedy, C. (2015). Batch normalization: Accelerating deep network training by reducing internal covariate shift. *arXiv preprint arXiv:1502.03167*.

Karpathy, A., Johnson, J., & Fei-Fei, L. (2015). Visualizing and understanding recurrent networks. *arXiv preprint arXiv:1506.02078*.

Li, H., Xu, Z., Taylor, G., Studer, C., & Goldstein, T. (2018). *Visualizing the loss landscape of neural nets.* Paper presented at the Advances in neural information processing systems.

Pascanu, R., Mikolov, T., & Bengio, Y. (2013). *On the difficulty of training recurrent neural networks.* Paper presented at the International conference on machine learning.

Rumelhart, D. E., Hinton, G. E., & Williams, R. J. (1985). *Learning internal representations by error propagation*. Retrieved from

Rumelhart, D. E., Hinton, G. E., & Williams, R. J. (1986). Learning representations by back-propagating errors. *Nature, 323*(6088), 533-536.

Sagheer, A., & Kotb, M. (2019a). Time series forecasting of petroleum production using deep LSTM recurrent networks. *Neurocomputing, 323*, 203-213.

Sagheer, A., & Kotb, M. (2019b). Unsupervised pre-training of a Deep LStM-based Stacked Autoencoder for Multivariate time Series forecasting problems. *Scientific reports, 9*(1), 1-16.

Santurkar, S., Tsipras, D., Ilyas, A., & Madry, A. (2018). *How does batch normalization help optimization?* Paper presented at the Advances in neural information processing systems.

Schuster, M., & Paliwal, K. K. (1997). Bidirectional recurrent neural networks. *IEEE transactions on Signal Processing, 45*(11), 2673-2681.

Siami-Namini, S., Tavakoli, N., & Namin, A. S. (2019). A comparative analysis of forecasting financial time series using arima, lstm, and bilstm. *arXiv preprint arXiv:1911.09512*.

Sra, S., Nowozin, S., & Wright, S. J. (2012). *Optimization for machine learning*: Mit Press.

Srivastava, N., Mansimov, E., & Salakhudinov, R. (2015). *Unsupervised learning of video representations using lstms.* Paper presented at the International conference on machine learning.

Sutskever, I., Martens, J., Dahl, G., & Hinton, G. (2013). *On the importance of initialization and momentum in deep learning.* Paper presented at the International conference on machine learning.

Wu, Y., Yuan, M., Dong, S., Lin, L., & Liu, Y. (2018). Remaining useful life estimation of engineered systems using vanilla LSTM neural networks. *Neurocomputing, 275*, 167-179.

Zaroug, A., Lai, D. T., Mudie, K., & Begg, R. (2020). Lower Limb Kinematics Trajectory Prediction Using Long Short-Term Memory Neural Networks. *Frontiers in bioengineering and biotechnology, 8*.
